# Costs of major intracranial, gastrointestinal and other bleeding events in patients with atrial fibrillation – a nationwide cohort study

**DOI:** 10.1186/s12913-017-2331-z

**Published:** 2017-06-12

**Authors:** Marie Jakobsen, Christophe Kolodziejczyk, Eskild Klausen Fredslund, Peter Bo Poulsen, Lars Dybro, Søren Paaske Johnsen

**Affiliations:** 1 KORA, Danish Institute for Local and Regional Government Research, Købmagergade 22, DK-1150 København K, Denmark; 2 Pfizer Denmark, Lautrupvang 8, DK-2750 Ballerup, Denmark; 3 Department of Clinical Epidemiology, Aarhus University Hospital, Olofs Palmes Allé 43-45, DK-8200 Aarhus N, Denmark

**Keywords:** Anticoagulants, Atrial fibrillation, Bleeding, Cost of illness, Costs, Propensity score matching

## Abstract

**Background:**

Use of oral anticoagulation therapy in patients with atrial fibrillation (AF) involves a trade-off between a reduced risk of ischemic stroke and an increased risk of bleeding events. Different anticoagulation therapies have different safety profiles and data on the societal costs of both ischemic stroke and bleeding events are necessary for assessing the cost-effectiveness and budgetary impact of different treatment options. To our knowledge, no previous studies have estimated the societal costs of bleeding events in patients with AF.

The objective of this study was to estimate the 3-years societal costs of first-incident intracranial, gastrointestinal and other major bleeding events in Danish patients with AF.

**Methods:**

The study was an incidence-based cost-of-illness study carried out from a societal perspective and based on data from national Danish registries covering the period 2002-2012. Costs were estimated using a propensity score matching and multivariable regression analysis (first difference OLS) in a cohort design.

**Results:**

Average 3-years societal costs attributable to intracranial, gastrointestinal and other major bleeding events were 27,627, 17,868, and 12,384 EUR per patient, respectively (2015 prices). Existing evidence shows that the corresponding costs of ischemic stroke were 24,084 EUR per patient (2012 prices). The average costs of bleeding events did not differ between patients with AF who were on oral anticoagulation therapy prior to the event and patients who were not.

**Conclusions:**

The societal costs attributable to major bleeding events in patients with AF are significant. Intracranial haemorrhages are most costly to society with average costs of similar magnitude as the costs of ischemic stroke. The average costs of gastrointestinal and other major bleeding events are lower than the costs of intracranial haemorrhages, but still substantial. Knowledge about the relative size of the costs of bleeding events compared to ischemic stroke in patients with AF constitutes valuable evidence for decisions-makers in Denmark as well as in other countries.

**Electronic supplementary material:**

The online version of this article (doi:10.1186/s12913-017-2331-z) contains supplementary material, which is available to authorized users.

## Background

Over 6 million Europeans currently suffer from atrial fibrillation (AF), and projections suggest that the prevalence of AF will at least double by 2050 due to the aging population [1–3]. The primary clinical significance of AF lies in an increased risk of ischemic stroke [4]. Making clinical decisions about the use oral anticoagulation therapy in patients with AF involves a trade-off as oral anticoagulation therapy reduces the risk of ischemic stroke but may increase the risk of bleeding events [5–8]. Evidence suggests that the incidence of bleeding events associated with oral anticoagulation therapy has been on the rise in developing countries [9–11].

Estimates of the societal costs of both ischemic stroke and bleeding events in patients with AF are necessary for assessing the cost-effectiveness and budgetary impact of different treatment options, including oral anticoagulation therapy versus no therapy and various oral anticoagulation therapies with different safety profiles. A recent Danish registry study found that the average 3-years societal costs of first-incident ischemic stroke in patients with AF are 24,084 EUR per patient (2012 prices), including direct costs related to healthcare and social care services and indirect costs due to production lost to society [12]. To our knowledge, no previous studies have estimated the societal costs of bleeding events (intracranial, gastrointestinal and other) in patients with AF, including both direct and indirect costs. A number of studies have investigated the costs of bleeding events in the general population [13–16]. However, cost estimates of bleeding events in the general population may differ from the costs of bleeding events in patients with AF as evidence suggests that bleeding events associated with oral anticoagulant therapy are generally more severe [17].

The aim of this study was to estimate the 3-years societal costs of first-incident intracranial, gastrointestinal and other major bleeding events in Danish patients with AF, including costs of healthcare, social care services and production lost to society. A subgroup analysis was performed for patients with AF who were on oral anticoagulation therapy prior to the bleeding event.

## Methods

This study was an incidence-based cost-of-illness study carried out from a societal perspective and based on data from national registries covering the entire Danish population. Every Danish citizen has a permanent personal registration number that enables linkage between registries at the individual level. The costs attributable to intracranial, gastrointestinal and other bleeding events in patients with AF were estimated in a cohort design using propensity score matching and multivariable regression analysis (first difference OLS (Ordinary Least Squares)) [18].

### Study population

The source population was identified from the National Patient Registry as all patients who were hospitalised in the period 1994-2012 with AF as the primary or secondary diagnosis (ICD 10: I48) [19]. The overarching ICD 10 code I48 covering both atrial fibrillation and atrial flutter was used since most patients with atrial flutter also have (episodes of) atrial fibrillation and misclassification is likely to be substantial. Furthermore, the risk of stroke and peripheral embolism are comparable for patients with atrial fibrillation and atrial flutter and European and North American guidelines do not distinguish between the two. Other Danish registry-based studies have also used the overarching ICD code I48 to identify patients with atrial fibrillation [20–22].

**Fig. 1:**
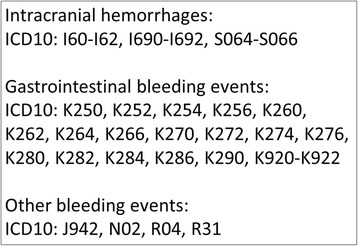
Bleeding diagnoses (ICD 10) included in the study

**Fig. 2:**
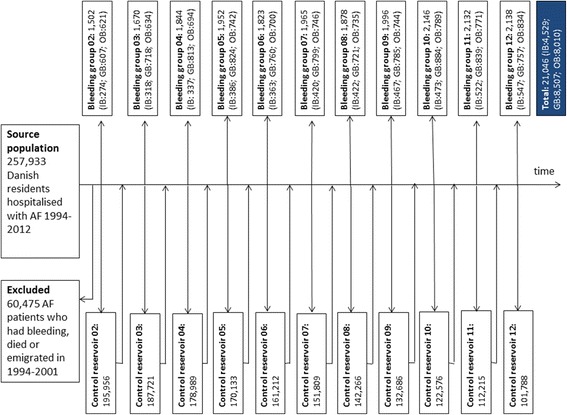
Flow chart

From the source population, the bleeding groups (exposed) were identified for each year during the period from January 1st 2002 till December 31st 2012 as patients who were hospitalised in the year in question with an ICD 10 bleeding diagnosis as the primary or secondary diagnosis following or at the same time as the AF diagnosis (Figs. 1 and 2). Patients were excluded if they had been hospitalised with a bleeding event during the period 1994-2001 to delimit the analysis to incident cases only. To each bleeding group, potential controls (the control reservoir of non-exposed) were identified as the remaining AF patients in the source population and propensity score matching was used to select controls from the control reservoir. The propensity score was based on the following matching criteria chosen to identify clinically comparable groups: Age, sex, co-morbidity (measured by the Charlson index), and risk of bleeding events (measured by the HAS-BLED score) at baseline.

All patients were censored at death. Furthermore, patients were censored from the control group if they were hospitalised with the type of bleeding event in question as either the primary or secondary diagnosis after the incidence year (from this year on, these patients were assigned to the bleeding group to avoid contamination of the control group). Patients in the control group assigned to patients with intracranial haemorrhages were not censored from this control group if they were hospitalised with gastrointestinal or other bleeding events. Similarly, patients in the control group assigned to patients with gastrointestinal and other bleeding events were not censored from these control groups if they were hospitalised with intracranial/other bleeding events or intracranial/gastrointestinal bleeding events, respectively.

Patients with AF on oral anticoagulant therapy were identified from the Registry of Medical Products Statistics (Prescription Registry) as patients who, in the year before the bleeding event, had redeemed at least two prescriptions with the ATC codes B01AA (vitamin k antagonists (VKA)) or B01AE07 (dabigatran) which were the only oral anticoagulation therapies on the Danish market before 2012 [23]. The study did not have sufficient power to distinguish between VKA and dabigatran as dabigatran had only been on the Danish market since 2011.

### Costs

The average annual costs attributable to bleeding events were estimated over a 3-years period from the year in which the bleeding event occurred (the incidence year) and onwards using the matched control/regression approach which is considered the gold standard method of cost-of-illness studies [24].

Firstly, standardised costs were calculated for patients in the bleeding groups and control groups as costs in year t (*t* = 0, 1, 2) after the incidence year minus the costs incurred in the year before the bleeding event (the baseline year). Costs incurred in the baseline year were deducted as it was not possible to isolate costs according to diagnosis when including costs outside hospitals. Furthermore, patients were assumed to consume healthcare and social care resources even if they had not experienced the bleeding event as the majority of the study population was elderly with other comorbidities.

Secondly, attributable costs were calculated as standardised costs incurred by patients in the bleeding group minus standardised costs incurred by patients in the control group:

$$ {Atributable\  costs}_t^i=\left({C}_t^d\colorbox[rgb]{1,1,0}{$-$}{C}_{-1}^d\right)-\left({C}_t^c-{C}_{-1}^c\right) $$,

where C = costs, d = bleeding group, c = control group, and t = year 0, 1 and 2.

In this way, the cost estimates were adjusted for changes in costs from the baseline year and onwards that were unlikely to be related to the bleeding event.

Healthcare costs included hospital costs, costs of visits to general practitioners (GPs) and other healthcare professionals in the primary care setting, and costs of prescription medicine. The Danish healthcare system is public and mainly tax-financed. Hospital services and services provided by GPs and specialist physicians in the primary care setting are free of charge. Services provided by other healthcare professionals outside hospitals and prescription medicine are reimbursed by the National Health Insurance with a patient co-payment added. Data on hospital costs were collected from the National Patient Registry [19]. Data on costs of visits to GPs and other healthcare professionals in the primary care setting were collected from the National Health Insurance Registry [25]. Both the National Patient Registry and the National Health Insurance Registry are used for payment purposes, and the quality of the data in these registries are considered to be of high. Hospital resource use and visits to GPs and other healthcare professionals in the primary care setting were priced according to the actual tariffs for the specific healthcare services provided. Data on costs of prescription medicine dispensed from Danish pharmacies were collected from the Prescription Registry [23]. The Prescription Registry is considered to be complete as Danish pharmacies have exclusive right to sell prescription medicine. The costs of prescription medicine were estimated using the pharmacy selling price (PSP), including both the share covered by the National Health Insurance and the patient co-payment [26].

Costs of social care services included costs of home help and nursing homes that are financed by the municipalities in Denmark. Data on home help were obtained from Statistics Denmark (the Elderly Care Documentation) and data on use of nursing homes were obtained from the Building Regulations Register linked to the Civil Registration System [27]. The costs of home help and nursing homes were estimated using average tariffs.

Estimation of the production lost to society was based on the human capital approach which is a commonly used method for valuation of indirect costs assuming that the production lost to society is equal to the value of lost earnings [28, 29]. The value of lost earnings was estimated using data from Statistics Denmark on income from employment. Only lost earnings of patients who were 18-65 years of age were included (the official retirement age in Denmark was 65 in 2012). Lost earnings of informal caregivers were not included.

The average annual attributable costs in year t (*t* = 0, 1, 2) after the incidence year were estimated for each bleeding group. Costs of social care services and production lost to society were estimated for the bleeding groups 2002-2012 and healthcare costs were estimated for the bleeding groups 2002-2011 as data on hospital tariffs were only available until 2011. The average annual attributable costs reported here are average costs across bleeding groups, i.e. the estimated costs in the incidence year cover the bleeding groups 2002-2012 whereas the estimated costs in year 1 and 2 after the bleeding event cover the bleeding groups 2002-2011 and 2002-2010, respectively.

All costs were inflated to 2015 prices and converted to euros (EUR) based on the average exchange rate in 2015 (100 EUR = 746 DKK). The present value of the 3-years costs was calculated using a discount rate of 4% as currently recommended by the Danish Ministry of Finance [30].

### Covariates

The Charlson index and HAS-BLED score were included as covariates to adjust for differences between the bleeding and control groups with regard to comorbidity and risk of bleeding events, respectively [31, 32]. The Charlson index was calculated using information from the National Patient Registry on diagnoses related to admissions and outpatient visits to hospitals 5 years before the incidence year. The HAS-BLED score was calculated using information from the National Patient Registry and the Prescription Registry on patients’ age, bleeding history, history of stroke, hypertension, abnormal renal or liver function, and drug consumption or alcohol abuse in the baseline year.

Furthermore, age, sex, region of residence, and socioeconomic status measured by education, employment, and income were included as covariates. Data on these variables were obtained from The Civil Registration System as well as the Education Register, the Integrated Database for Labour Market Research, and the Income Register of Statistics Denmark [33–35].

### Statistical analysis

Nearest neighbour propensity score matching with replacement was used to select four controls for each patient in the bleeding groups [36]. Matching with replacement can often decrease bias compared to matching without replacement because controls who are good matches to more than one individual in the exposed group can be used multiple times [37]. The majority of the controls in this study are used only one or two times (cf. Additional file 1: Figure S1 provided as supplementary material).

An absolute standardised difference below 10% and a variance ratio between 0.8 and 1.25 were considered to support the assumption of balance between groups [38].

Average costs attributable to intracranial, gastrointestinal and other major bleeding events were estimated in a differences-in-differences regression model as β_1_ (first difference OLS):

$$ \Delta {C}_t^i={\beta}_0+{\beta}_1{D}_i+{\beta}_2{X}_{1 i}+{\beta}_3{X}_{2 i}+\dots +{\varepsilon}_i $$,

where ${\Delta \mathrm{C}}_{\mathrm{t}}^{\mathrm{i}}={C}_t^i-{C}_{-1}^i$, D_i_ = dummy variable indicating whether the patient belongs to the bleeding or the control group, X_i_ = covariates (see Table 1), and t = year 0, 1 and 2.

**Table 1:** Baseline characteristics of the study population. The table shows baseline characteristics of patients in different bleeding groups and the matched controls

Characteristics	Intracranial haemorrhages		Gastrointestinal bleeding events		Other bleeding events	
	Bleeding group	Control group	Bleeding Group	Control group	Bleeding group	Control group
Female sex (%)	45%	45%	49%	50%	29%	29%
Age, average (years)	77 years	77 years	78 years	78 years	76 years	76 years
Age group (%)						
< 65	11%	11%	10%	10%	13%	13%
65-74	23%	23%	21%	21%	25%	25%
≥ 75	66%	66%	69%	69%	62%	62%
Charlton index (%)						
= 0	47%	48%	43%	43%	44%	44%
= 1-2	39%	39%	40%	40%	40%	40%
≥ 2	14%	13%	17%	17%	16%	16%
HAS-BLED score, average	2.43	2.43	2.55	2.55	2.48	2.48
Education (%)						
Primary and lower secondary school	41	43	42	41	40	40
Higher secondary school and vocational training	27	26	24	24	29	28
Higher education	13	12	9	11	12	13
Missing	19	19		24	19	19
Labour market affiliation (%)						
Wage earner	5%	6%	4%	5%	6%	7%
Self-employed	3%	3%	2%	3%	4%	4%
Unemployed	1%	0%	1%	1%	1%	1%
Retired	89%	88%	92%	89%	87%	86%
Other	0%	0%	0%	0%	0%	0%
Missing	1%	2%	1%	1%	1%	2%
Annual income, average (EUR)	29,619 EUR	29,638 EUR	29,995 EUR	29,703 EUR	30,069 EUR	32,023 EUR

The statistical significance of cost estimates were evaluated using a two-sided t-test (H_0_: μ = 0 against the hypothesis H_1_: μ ≠ 0). This was equivalent to testing whether average standardised costs per patient in the bleeding group were significantly different from average standardised costs per patient in the control group. The statistical significance level was set at *P* < 0.05. A possible skewed distribution of costs was not considered a problem in this study due to the large sample size [39].

Sensitivity analyses were performed to investigate the robustness of the healthcare cost estimates. It was analysed how the cost estimates were affected if only patients who had been hospitalised with a bleeding event as the primary diagnosis were included. Moreover, the consequences of including only patients who survived the incidence year and selecting another baseline year were investigated. Finally, cost estimates beyond the 3-years time horizon were examined.

Data were assumed to be missing at random and the statistical analyses were carried out using available data only (data were missing for less than 1% of the observations).

Analyses were performed using the statistical package SAS 9.3 for Windows (SAS Institute, Cary, NC, USA) and Stata 13.1 (StataCorp, College Station, TX).

## Results

In total, 21,046 patients with AF were hospitalised with a first-incident bleeding event during the period 2002-2012. Of these, 4529 patients experienced intracranial haemorrhages while 8507 and 8010 patients experienced gastrointestinal and other major bleeding events, respectively.

Table 1 shows background characteristics of the patients in the three bleeding groups and the matched controls. There were no significant imbalances between the bleeding and control groups with regard to the covariates used in the propensity score matching (cf. Additional files 2, 3, 4: Figures S2, S3, S4 provided as supplementary material). In general, imbalances for other covariates were also within the boundaries considered to support the assumption of balance between groups.

The estimated average 3-years societal costs attributable to intracranial haemorrhages were 27,627 EUR per patient (present value calculated in the incidence year and stated in 2015 prices) corresponding to 18,049 EUR in the incidence year, 6922 EUR in the year after, and 4356 EUR 2 years after the event when not discounted to present value (Table 2). The estimated average 3-years societal costs attributable to gastrointestinal and other major bleeding events were 17,868 EUR and 12,384 EUR, respectively (Table 3- 4).

**Table 2:** Intracranial haemorrhages – average attributable costs per patient, EUR (2015 prices)

	3-years costs (present value calculated in the incidence year)^a^	Annual attributable cost estimates (not discounted)^b^		
		Year 0 (incidence year)	Year 1 after the bleeding event	Year 2 after the bleeding event
Direct costs				
Healthcare	18,061	16,309	2985	−427
Inpatient hospital care	18,078	16,341***	3094***	−557
Outpatient hospital care	−49	132	−192	2
Private practice health prof.	73	−69***	68**	86**
Prescribed medicine	−42	−96***	16	41
Social care services	7524	1546	3313	3345
Home help	3929	419**	1844***	2048***
Nursing home	3595	1127***	1469***	1297***
Indirect costs				
Productivity loss	2042	194	624**	1438***
Direct and indirect costs	27,627	18,049	6922	4356

^a^3-years costs (present value) are equal to the sum of the costs in year 0 and the discounted value of the costs in years 1 and 2 after the bleeding event

^b^T-tests were used to investigate whether the annual attributable cost estimates related to hospital care, visits to GPs and other health professionals in the primary care setting, prescribed medicine, home help, nursing home and productivity loss were significantly different from zero (H_0_: μ = 0 against the hypothesis H_1_: μ ≠ 0.). Asterisks indicate that the null-hypothesis was rejected (*p < 0.05, **p < 0.01, ***p < 0.001)

**Table 3:** Gastrointestinal bleeding events – average attributable costs per patient, EUR (2015 prices)

	3-years costs (present value calculated in the incidence year)^a^	Annual attributable cost estimates (not discounted)^b^		
		Year 0 (incidence year)	Year 1 after the bleeding event	Year 2 after the bleeding event
Direct costs				
Healthcare	14,492	12,736	1796	658
Inpatient hospital care	13,453	12,090***	1521***	475
Outpatient hospital care	852	516***	249**	142
Private practice health prof.	−4	79***	−42***	−46**
Prescribed medicine	190	51***	68***	88***
Social care services	3060	851	1138	1339
Home help	1840	437***	696***	874***
Nursing home	1219	414**	442**	465*
Indirect costs				
Productivity loss	317	115	160	66
Direct and indirect costs	17,868	13,702	3093	2063

^a^See Table 2

^b^See Table 2

Asterisks indicate that the null-hypothesis was rejected (*p < 0.05, **p < 0.01, ***p < 0.001)

**Table 4:** Other bleeding events – average attributable costs per patient, EUR (2015 prices)

	3-years costs (present value calculated in the incidence year)^a^	Annual attributable cost estimates (not discounted)^b^		
		Year 0 (incidence year)	Year 1 after the bleeding event	Year 2 after the bleeding event
Direct costs				
Healthcare	9048	8393	1035	23
Inpatient hospital care	7418	7192***	640*	−100
Outpatient hospital care	1492	1017***	401***	162
Private practice health prof.	83	136***	−8	−46**
Prescribed medicine	54	48***	3	7
Social care services	2119	852	790	640
Home help	1364	597***	501***	367*
Nursing home	755	255*	289*	273
Indirect costs				
Productivity loss	1218	281	419	631
Direct and indirect costs	12,384	9526	2244	1294

^a^See Table 2

^b^See Table 2

Asterisks indicate that the null-hypothesis was rejected (*p < 0.05, **p < 0.01, ***p < 0.001)

Healthcare costs accounted for 66% of total 3-years societal costs attributable to intracranial haemorrhages while social care costs and production lost to society accounted for 27 and 7%, respectively. For gastrointestinal and other major bleeding events, healthcare costs accounted for 81 and 73%, respectively. For all three types of bleeding events, the majority of the healthcare costs were due to hospitalisations in the incidence year. One year after an intracranial haemorrhage and 2 years after gastrointestinal and other major bleeding events, average social care costs exceeded average healthcare costs.

**Fig. 3:**
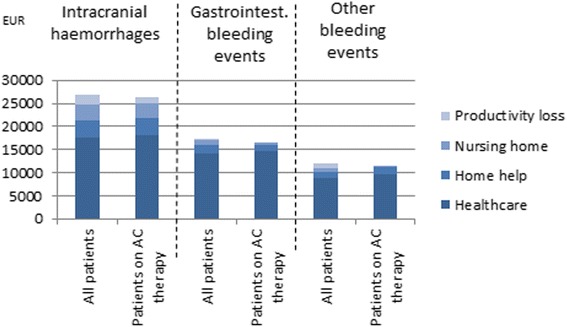
Three-years costs of bleeding events in AF patients on anticoagulation (AC) therapy, EUR (2015 prices)

The cost estimates remained essentially unchanged when estimated for the subgroup of patients with AF who were on oral anticoagulant therapy prior to the bleeding event (Fig. 3).

Sensitivity analyses showed that the 3-years healthcare cost estimates were relatively robust (Table 5). When only patients with AF who were hospitalised with a bleeding event as the primary diagnosis were included, the healthcare cost estimates decreased for gastrointestinal and other major bleeding events. This indicates that patients who are hospitalised with gastrointestinal and other major bleeding events as the secondary diagnosis generally have other more complicated diseases that increase costs. The healthcare cost estimates remained essentially unchanged for intracranial haemorrhages since only few patients were hospitalised with intracranial haemorrhage as the secondary diagnosis. The healthcare cost estimates increased for intracranial haemorrhages when only patients who were alive after the incidence year were included whereas the cost estimates for gastrointestinal and other bleeding events remained essentially unchanged. The difference was due to a higher mortality rate among patients with intracranial haemorrhages increasing the costs per surviving patient (cf. Additional file 5: Figure S5 provided as supplementary material). The cost estimates for all three types of bleeding events increased when the baseline year was 2 years before the incidence year as average costs incurred by patients in the bleeding groups were increasing in the years leading up to the bleeding event. The cost estimates decreased if the time horizon was prolonged to 5 and 10 years as the average standardised costs of patients in the control groups generally exceeded average standardised costs of patients in the bleeding groups from 3 years after the bleeding event and henceforth. Focus in this study has been on the 3-years costs as the comparability between the bleeding and the control groups decreased over time due to differences in mortality.

**Table 5:** Results of the sensitivity analysis

Scenario	Attributable healthcare costs, EUR (2015 prices) (3-years present value unless otherwise stated)		
	Intracranial hemorrhages	Gastrointestinal bleeding events	Other bleeding events
Base scenario	18,061	14,492	9048
Only patients with bleeding as primary diagnosis	17,540	12,016	6481
Only patients who survive the incidence year	23,439	14,595	8649
Baseline year 2 years before the incidence year	18,925	16,934	11,379
5-years time horizon	16,279	13,534	8000
10-years time horizon	4302	3558	392

## Discussion

The present study found that the average 3-years societal costs attributable to intracranial, gastrointestinal and other major bleeding events in patients with AF were 27,627 EUR, 17,868 EUR, and 12,384 EUR per patient, respectively (2015 prices). Existing evidence shows that the corresponding costs attributable of ischemic stroke are 24,084 EUR per patient (2012 prices, 100 EUR = 128 USD in 2012) [12]. The average costs of bleeding events did not differ between patients with AF who were on oral anticoagulation therapy prior to the event and patients who were not.

This study has a strong methodological design using an incidence-based case-control approach and propensity score matching to minimise the risk of confounding by age, sex, co-morbidity, bleeding risk, and socio-economic characteristics. Furthermore, the use of national registry data reduces the risk of selection and information bias as the registries cover the entire Danish population, and as data are prospectively registered and the quality is considered to be high. Another strength involves the use of a large data set covering a 10-years period, making the cost estimates more robust. The inclusion of social care costs related to home help and nursing homes, additional to healthcare costs, is another important strength of this study. Social care costs related to rehabilitation, assistive devices, home modifications, and informal care were not included as data were not available from the national registries. The cost estimates therefore remain conservative.

The study also has a number of limitations. Firstly, the cost estimates concern major bleeding events as only patients who had been hospitalised with bleeding events as the primary or secondary diagnosis were included in the study. Patients with minor bleeding events who were not hospitalised were probably less costly.

Secondly, only patients who had been hospitalised in the period 1994-2012 with AF as the primary or secondary diagnosis were included in the source population of the study from which both cases and controls were drawn. Thus, the study estimates the costs of bleeding events among patients with severe AF/comorbidity. The estimated costs would probably have been higher if all patients with AF had been included in the source population due to lower costs in the control group. However, the number of patients with AF not registered in hospital records is low in Denmark as national clinical guidelines recommend evaluation in hospital.

Thirdly, there is a risk of confounding due to the observational nature of the study. The risk of confounding related to observable background characteristics was minimized by a well-balanced propensity score-matched and multivariable regression design. However, the risk of confounding increased over time after the bleeding event as the comparability between the bleeding and control groups diminished due to differences in mortality (cf. Additional file 5: Figure S5 provided as supplementary material). Therefore, the study was delimited to a 3-years time horizon.

It is possible that some of the differences in costs between the bleeding and control groups in this study were attributable to ischemic stroke. However, as a differences-in-differences approach was used and as the incidence of ischemic stroke in the bleeding and control groups was similar, this problem is not regarded a major concern. Overall, 16% of the patients in the bleeding groups had an ischemic stroke during the observation period 1994-2012 compared to 12% in the matched control groups. In the bleeding group, 6% had a stroke the same year as the bleeding event (*t* = 0), 4% had had a previous stroke, and 6% had a stroke in the years after the bleeding events (the follow-up period). In the control groups, 12% had a stroke during the follow-up period. The fraction of patients in the control groups who had a stroke in the incidence year (*t* = 0) or earlier was below 0.5%. Thus, the incidence of ischemic stroke was 12% in both the bleeding groups and the control groups from the incidence year (*t* = 0) and onwards.

Fourthly, the validity of the cost estimates depends on the accuracy of the information in the Danish registries. The risk of misclassification remains even though the quality of data in the Danish registries is high. Most importantly, the ICD-10 coding of AF and bleeding events in the National Patient Registry may not be entirely accurate. However, validation studies have reported high predictive values [40, 41]. Moreover, the Building Regulation Registry, which was used to estimate costs of nursing homes, may contain errors as it is not audited. Since possible misclassification is most likely non-differential, this may have biased the results in a conservative direction.

Furthermore, use of healthcare resources was valued according to tariffs that may not reflect the true opportunity costs. However, tariffs were the best available approximation of actual costs at national level in this study.

Production lost to society was valued according to the human capital approach where the production lost to society (the productivity loss) is assumed to be equal to the present value of all lost future earnings until retirement from the labour market. The human capital approach has been criticised for not taking into account the existence of involuntary unemployment [42]. Another commonly used method for valuing productivity loss is the friction cost approach. According to this method, the productivity loss occur only in the ‘friction period’ until the sick worker is replaced by a healthy unemployed person [42]. The estimated productivity loss in this study would most likely have been lower if the friction cost approach had been used. However, the absolute difference was reduced because the vast majority of the study population had retired from the labour market. Furthermore, the indirect costs were probably underestimated in this study as only productivity loss of patients in relation to paid work were included. Other types of indirect costs with implications for the overall societal costs were not included, e.g. productivity loss of informal caregivers as well as productivity loss of patients in relation to unpaid work (housework, voluntary work, and caregiving services).

Finally, the lack of observable differences between cost estimates of patients with AF in general and cost estimates of patients with AF who were on oral anticoagulation therapy prior to the bleeding event could be due to inaccuracy related to the identification of the latter group. The cost estimates of patients with AF on oral anticoagulation therapy were expected to be higher as evidence suggests that bleeding events associated with oral anticoagulant therapy are generally more severe [17]. In the present study, patients on oral anticoagulation therapy were identified as patients who had redeemed at least two prescriptions of anticoagulation drugs in the year before the bleeding event. It is likely that a proportion of these patients were not compliant with the treatment in the baseline year and/or subsequent years. Furthermore, some patients might have been compliant with the treatment in the incidence year but not in the baseline year. Even if the average costs of bleeding events are the same for patients who were on oral anticoagulation therapy prior to the event and patients who were not, the total costs of bleeding events will increase with the number of patients on anticoagulant therapy if anticoagulant therapy increases the incidence of these events.

Only few studies on the costs of bleeding events in patients with AF have been published so far. The cost estimates of the present study are higher than the cost estimates of existing studies as these studies have focused on hospital costs only. Cotté et al. (2014) estimated the 2-years hospital costs of bleeding events in 61,582 patients with AF in France who were eligible for anticoagulation treatment based on data from the French national hospital claims system covering the period 2006-2008 [43]. Mean 2-years hospital costs per patient were 7331 EUR for intracranial haemorrhages, 3601 EUR for gastrointestinal bleeding events, and 3941 EUR for other bleeding events. Luengo-Fernandez et al. (2013) estimated acute average healthcare costs of intracerebral haemorrhages in patients with AF in the United Kingdom using data from a population-based study including 17 patients recruited in the period 2002-2007 [44]. Average healthcare costs in the first 90 days after the event amounted to 15,627 EUR (100 EUR = 68 GBP in 2007). McBride et al. (2009) estimated the mean annual hospital costs of 311 patients with AF in Germany based on a prospective observational cohort study conducted from September 2003 to December 2004 [45]. Average annual costs of hospital admissions due to gastrointestinal and other bleeding events were 1743 EUR and 1802 EUR, respectively. Furthermore, Fanikos et al. (2005) investigated major bleeding complications in 2460 patients managed by the Brigham and Women’s Hospital Anticoagulation Service in the USA from 2000 to 2003 [46]. Overall, 11 patients had 12 non-fatal major bleeding complications during the study period. Of the 12 bleeding events, 5 (42%) were intracranial. The average hospital costs associated with these bleeding events were 14,179 EUR (100 EUR = 113 USD in 2003) per patient.

## Conclusions

In conclusion, this study is the first study to estimate the societal costs attributable to intracranial, gastrointestinal and other major bleeding events in patients with AF, including both direct healthcare and social care costs and indirect costs due to production lost to society. This study documents that these costs are significant. Even though the cost estimates of this study may not be directly transferable to another country, the study does provide an indication of the relative size of the costs of bleeding events compared to the costs of ischemic stroke in patients with AF which is valuable evidence for decision-makers in every country. Intracranial haemorrhages are most costly to society with average costs of similar magnitude as the costs of ischemic stroke [12]. The average costs of gastrointestinal and other major bleeding events are lower than the costs of intracranial haemorrhages, but still substantial. This new evidence strengthens the foundation for healthcare decision-making and priority setting regarding the future treatment of patients with AF.

## Additional files

Additional file 1: Figure S1. Extent of replacement. The figure shows number of times each control is used as a match in the study. (TIFF 72 kb)

Additional file 2: Figure S2. Intracranial haemorrhages – standardised differences and variance ratios in covariates. The figure illustrates the quality of the matching between patients with intracranial haemorrhages and their matched controls. (PNG 895 kb)

Additional file 3: Figure S3. Gastrointestinal bleeding events – standardised differences and variance ratios in covariates. The figure illustrates the quality of the matching between patients with gastrointestinal bleeding events and their matched controls. (PNG 896 kb)

Additional file 4: Figure S4. Other bleeding events – standardised differences and variance ratios in covariates. The figure illustrates the quality of the matching between patients with other bleeding events and their matched controls. (PNG 895 kb)

Additional file 5: Figure S5. Kaplan Meier survival curves. The figure shows the percentage of patients in the bleeding and control groups who are alive at the beginning of each year. (TIFF 187 kb)

## Abbreviations

AC: Anticoagulation
AF: Atrial fibrillation
GPs: General practitioners
OLS: Ordinary least squares
PSP: Pharmacy selling price
VKA: Vitamin K antagonists
