# Erratum to: costs of major intracranial, gastrointestinal and other bleeding events in patients with atrial fibrillation – a nationwide cohort study

**DOI:** 10.1186/s12913-017-2616-2

**Published:** 2017-10-09

**Authors:** Marie Jakobsen, Christophe Kolodziejczyk, Eskild Klausen Fredslund, Peter Bo Poulsen, Lars Dybro, Søren Paaske Johnsen

**Affiliations:** 1 KORA, Danish Institute for Local and Regional Government Research, Købmagergade 22, DK-1150 København K, Denmark; 2 Pfizer Denmark, Lautrupvang 8, DK-2750 Ballerup, Denmark; 3 Department of Clinical Epidemiology, Aarhus University Hospital, Olofs Palmes Allé 43-45, DK-8200 Aarhus N, Denmark

## Erratum

Following publication of the original article [1], the authors asked for the first formula to be changed from.

*Atributable costs i t* = (*C d t* \colorbox[*rgb*]1,1,0$ − $*C d* − 1) − (*C c t* − *C c* − 1).

to

$$ Atributable\ {costs}_t^i=\left({C}_t^d-{C}_{-1}^d\right)-\left({C}_t^c-{C}_{-1}^c\right), $$

The original article has been corrected.
