# Supplementary figures and images for: Costs of major intracranial, gastrointestinal and other bleeding events in patients with atrial fibrillation – a nationwide cohort study
Source: BMC Health Serv Res. 2017 Jun 12;17:398. doi: 10.1186/s12913-017-2331-z (PMC5469002; doi:10.1186/s12913-017-2331-z)

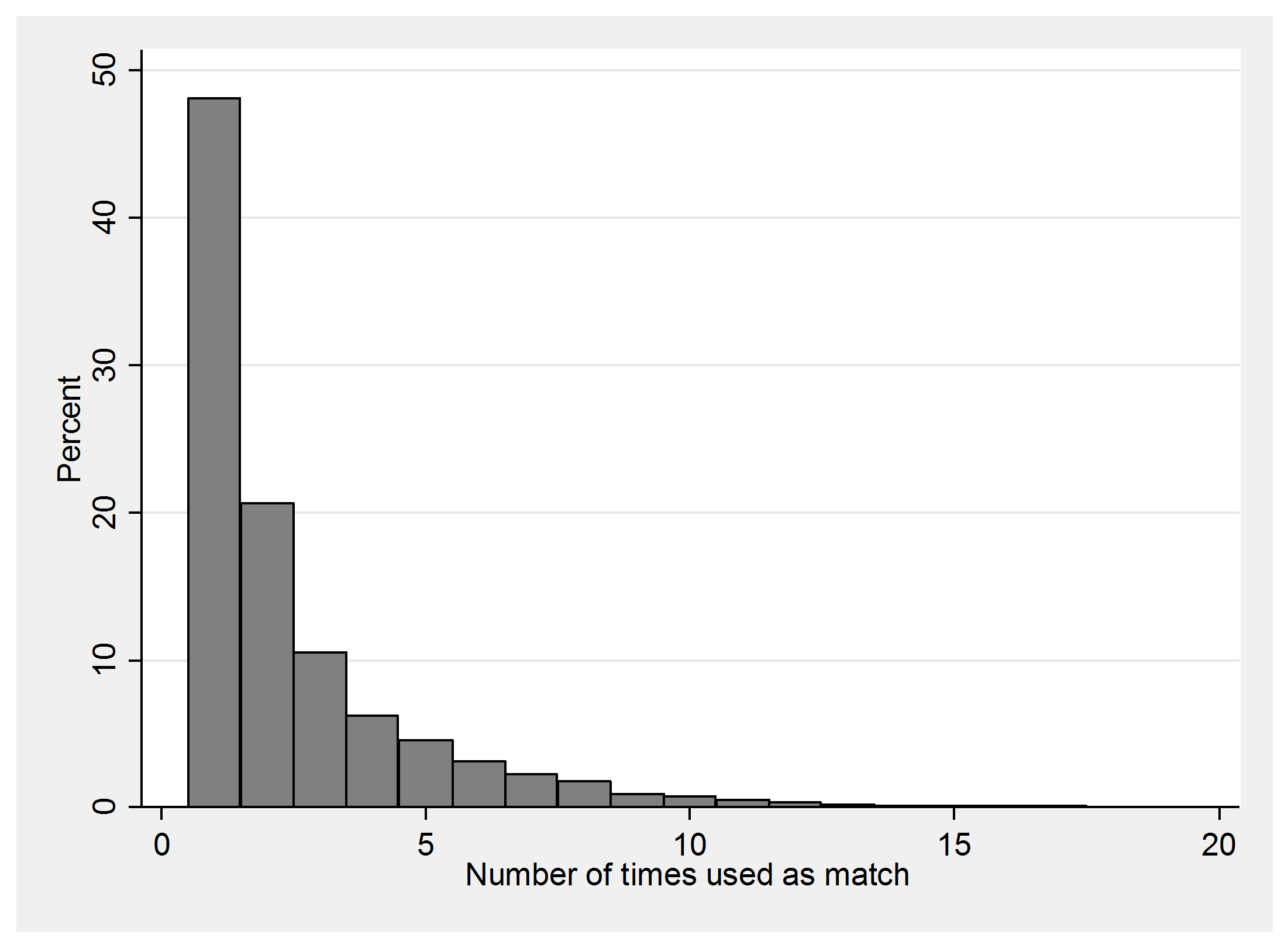

Supplement: Supplementary file 1 — Extent of replacement. The figure shows number of times each control is used as a match in the study. (TIFF 72 kb) [file 12913_2017_2331_MOESM1_ESM.tif]

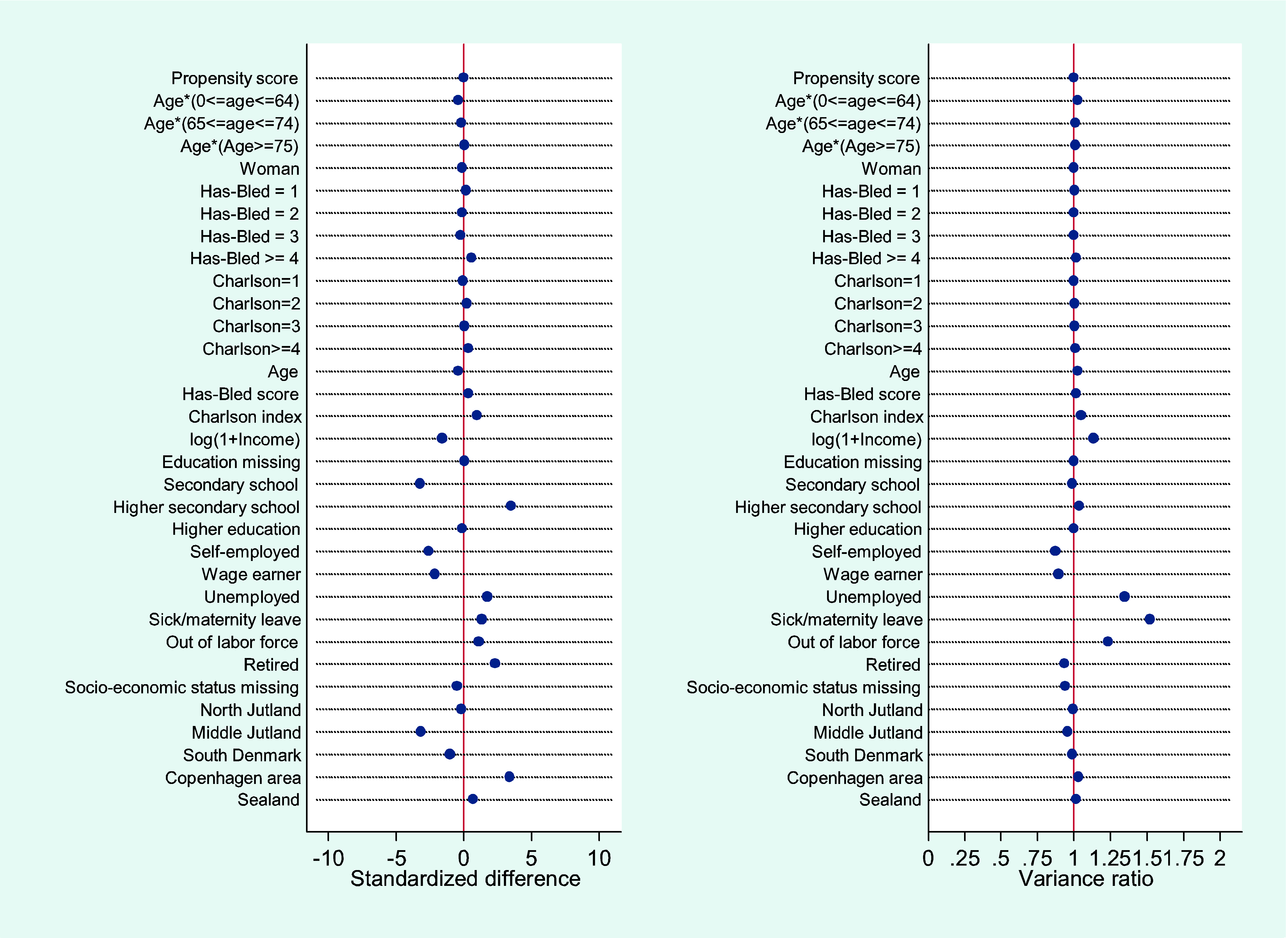

Supplement: Supplementary file 2 — Intracranial haemorrhages – standardised differences and variance ratios in covariates. The figure illustrates the quality of the matching between patients with intracranial haemorrhages and their matched controls. (PNG 895 kb) [file 12913_2017_2331_MOESM2_ESM.png]

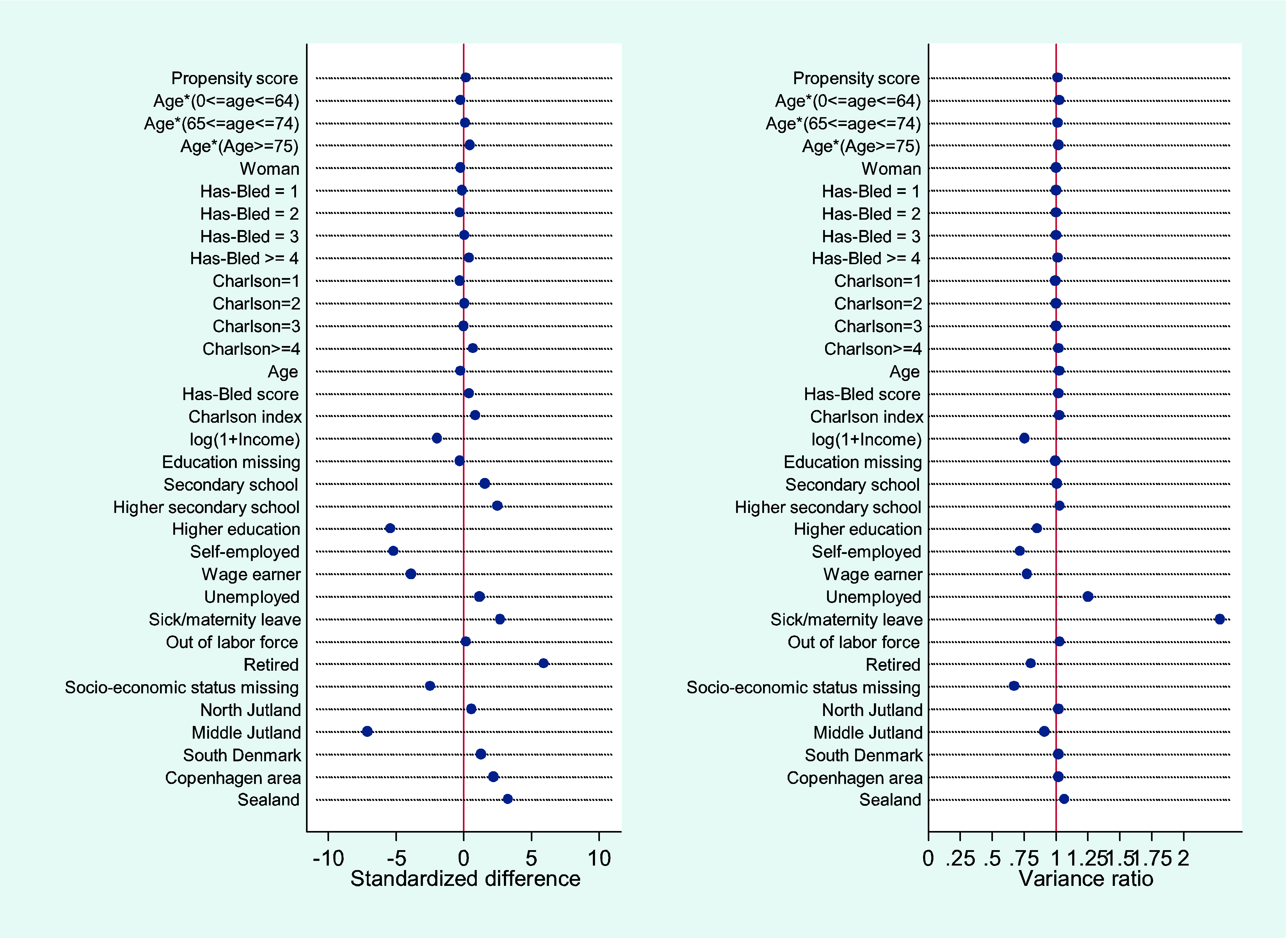

Supplement: Supplementary file 3 — Gastrointestinal bleeding events – standardised differences and variance ratios in covariates. The figure illustrates the quality of the matching between patients with gastrointestinal bleeding events and their matched controls. (PNG 896 kb) [file 12913_2017_2331_MOESM3_ESM.png]

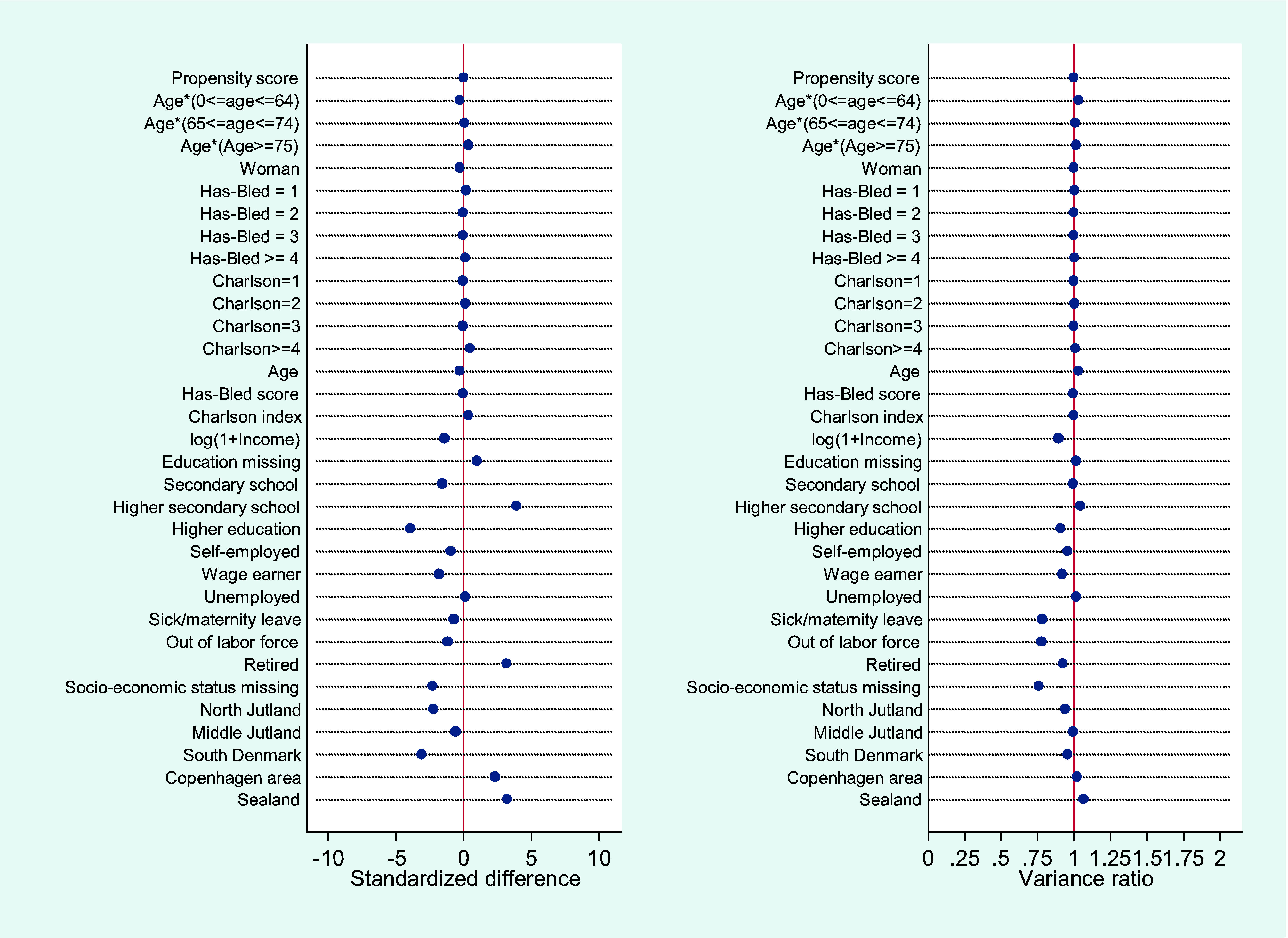

Supplement: Supplementary file 4 — Other bleeding events – standardised differences and variance ratios in covariates. The figure illustrates the quality of the matching between patients with other bleeding events and their matched controls. (PNG 895 kb) [file 12913_2017_2331_MOESM4_ESM.png]

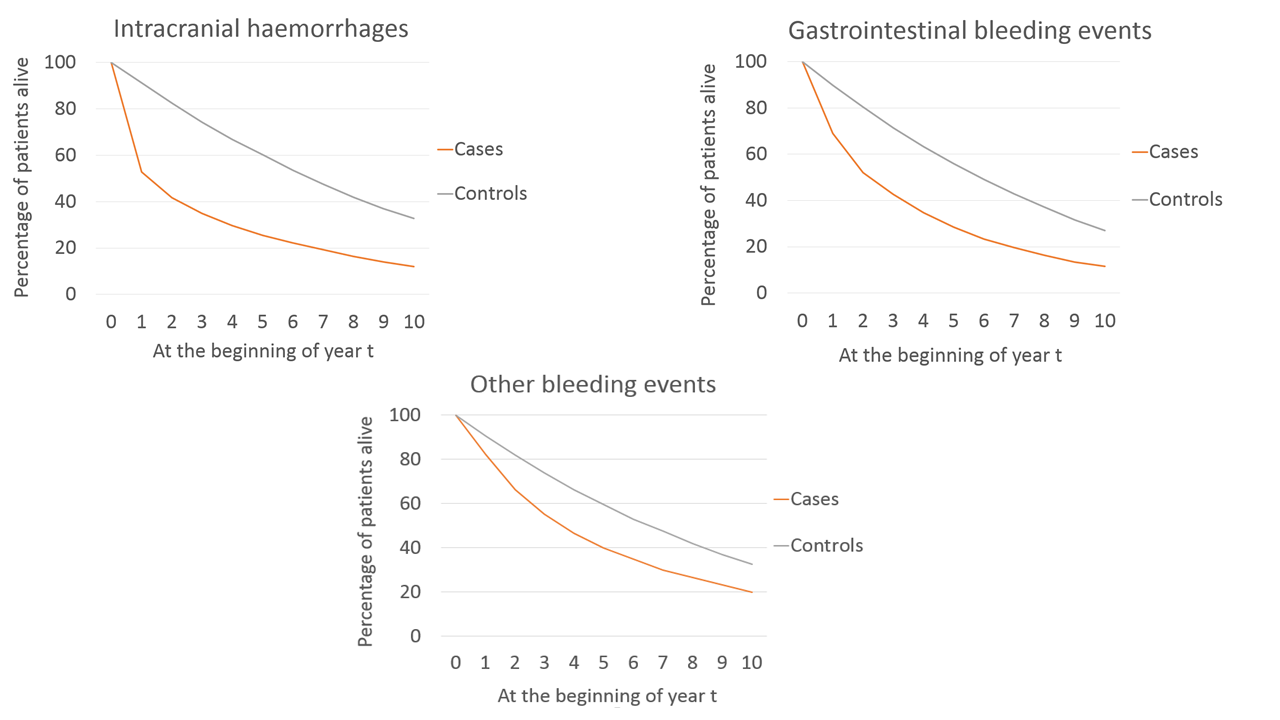

Supplement: Supplementary file 5 — Kaplan Meier survival curves. The figure shows the percentage of patients in the bleeding and control groups who are alive at the beginning of each year. (TIFF 187 kb) [file 12913_2017_2331_MOESM5_ESM.tif]
